# Evidence for a Conserved Function of Eukaryotic Pantothenate Kinases in the Regulation of Mitochondrial Homeostasis and Oxidative Stress

**DOI:** 10.3390/ijms24010435

**Published:** 2022-12-27

**Authors:** Camilla Ceccatelli Berti, Shalev Gihaz, Sonia Figuccia, Jae-Yeon Choi, Anasuya C. Pal, Paola Goffrini, Choukri Ben Mamoun

**Affiliations:** 1 Department of Chemistry, Life Sciences and Environmental Sustainability, University of Parma, 43124 Parma, Italy; 2 Department of Internal Medicine, Section of Infectious Diseases, Yale School of Medicine, New Haven, CT 06520, USA

**Keywords:** *Saccharomyces cerevisiae*, pantothenate kinase, vitamin B5, pantothenic acid, PKAN, model, genetic complementation

## Abstract

Human *PANK1*, *PANK2*, and *PANK3* genes encode several pantothenate kinase isoforms that catalyze the phosphorylation of vitamin B5 (pantothenic acid) to phosphopantothenate, a critical step in the biosynthesis of the major cellular cofactor, Coenzyme A (CoA). Mutations in the *PANK2* gene, which encodes the mitochondrial pantothenate kinase (PanK) isoform, have been linked to pantothenate-kinase associated neurodegeneration (PKAN), a debilitating and often fatal progressive neurodegeneration of children and young adults. While the biochemical properties of these enzymes have been well-characterized in vitro, their expression in a model organism such as yeast in order to probe their function under cellular conditions have never been achieved. Here we used three yeast mutants carrying missense mutations in the yeast PanK gene, *CAB1*, which are associated with defective growth at high temperature and iron, mitochondrial dysfunction, increased iron content, and oxidative stress, to assess the cellular function of human *PANK* genes and functional conservation of the CoA-controlled processes between humans and yeast. Overexpression of human *PANK1* and *PANK3* in these mutants restored normal cellular activity whereas complementation with *PANK2* was partial and could only be achieved with an isoform, PanK2^mtmΔ^, lacking the mitochondrial transit peptide. These data, which demonstrate functional conservation of PanK activity between humans and yeast, set the stage for the use of yeast as a model system to investigate the impact of PKAN-associated mutations on the metabolic pathways altered in this disease.

## 1. Introduction

**Figure 1:**
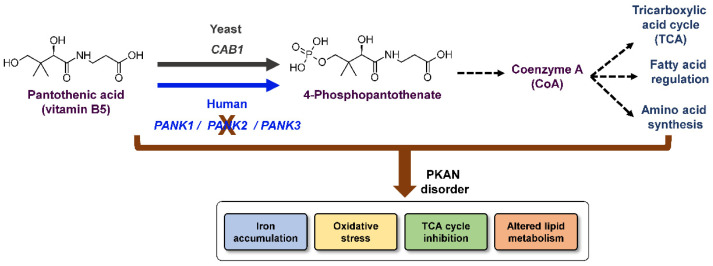
Pathway for CoA biosynthesis in humans and yeast and metabolic processes controlled by CoA. PanK enzymes catalyze the phosphorylation of pantothenic acid to form 4’-phosphopantothenate, which serves as a precursor for the biosynthesis of CoA. In yeast, PanK activity is catalyzed by the Cab1 enzyme, which is encoded by the *CAB1* gene. In humans, 4 PanK isoforms encoded by three *PANK* genes (*PANK1*, *2* and *3*) catalyze pantothenate phosphorylation. Mutations in the *PANK2* gene lead to major cellular and metabolic alterations, which ultimately result in the manifestations of the clinical symptoms of PKAN.

Coenzyme A (CoA) is an essential cofactor that plays a critical role in the operation of major cellular processes such as the citric acid cycle, lipid metabolism, redox regulation and ergosterol biosynthesis [1,2,3,4,5] (Figure 1). The synthesis of CoA initiates with the phosphorylation of pantothenic acid (vitamin B5) by a pantothenate kinase (PanK). In humans, this activity is catalyzed by PanK isoforms (1α, 1β, 2 and 3) encoded by three *PANK* genes, *PANK1*, *PANK2* and *PANK3* (Figure 1). 

A fourth *PANK* gene, *PANK4*, encodes a pseudo-enzyme with no kinase activity but with an active phosphatase domain [6,7]. PanK4 has recently reported to be involved in cellular signaling as a substrate of the PI3K effector kinase AKT [8]. The expression, activity, and co-regulation of human PanKs vary between tissues and cellular compartments and the importance of these differences in disease manifestation remain poorly understood [9]. PanK1 and PanK3 have been localized to the cytoplasm and nucleus [10,11,12] and their transcript levels were found to be high in the liver, kidney, brain and small intestine in the case of *PANK1*, and the brain, heart, thymus and skeletal muscle in the case of *PANK3* [13]. PanK2 is primarily found in the mitochondrial membranes with possible sub-localization to either the intermembranous space or cristae with high expression in neurons [10,11,12,14]. The importance of PanKs in human health was further highlighted by the discovery that mutations in the *PANK2* gene are associated with pantothenate kinase-associated neurodegeneration (PKAN). PKAN is a debilitating and fatal genetic disorder of children and young adults and for which no curative therapy exists [10,13,14,15,16]. This autosomal recessive disorder presents with dystonia, Parkinsonism, and is often accompanied with iron accumulation in the basal ganglia in the brain [13,17,18]. Epidemiological data suggest that PKAN accounts for 35 to 50% of all the neurodegenerative disorders associated with brain iron accumulation (NBIA disorders) [13,19]. While PKAN is classified into classical and atypical forms, the same mutation in the *PANK2* gene can be found in both patient populations suggesting that the molecular basis of the disease is more complex than presently presumed [13]. While the exact mechanism by which reduced or complete loss of PanK2 activity caused by missense or stop mutations, respectively, lead to disease presentation remains poorly understood, there is a clear link between PanK2 expression and mitochondrial dysfunction [20]. Studies in *Drosophila* and human cell lines support a model whereby a decrease in PanK2 expression and activity results in reduced levels of the phosphopantetheinyl-activated form of the mitochondrial acyl career protein (ACP). This partner molecule plays a critical role in mitochondrial biogenesis through the regulation of fatty acid synthesis (FASII pathway) as well as other important cellular and metabolic functions such as protein lipoylation and iron–sulfur cluster biogenesis [21,22]. This model is further supported by recent studies in mice lacking the *PANK2* gene, which showed altered mitochondrial respiratory function and defective iron homeostasis in the globus pallidus; molecular disruptions also seen in PKAN patients [15,16]. 

Unlike humans, the yeast *Saccharomyces cerevisiae* harbors a single gene, *CAB1*, which encodes PanK activity (Figure 1). The gene is essential for cell viability as its deletion results in cell death. Several *cab1* mutations have been generated and their impacts on enzyme activity and cell growth have been characterized [23]. Substitution of Glycine 351 to Serine was the first reported mutation in *CAB1* [1]. This mutation leads to a thermosensitive phenotype with yeast cells unable to grow at 37 °C [1]. Further characterization of this mutation showed altered growth on non-fermentable carbon sources, consistent with altered mitochondrial function and respiration [24], as well as increased susceptibility to ergosterol biosynthesis inhibitors [25]. Purified recombinant Cab1^G351S^ was found to have reduced pantothenate kinase activity (~10% that of the wild-type enzyme) [5]. Recently, a yeast model for PKAN, namely yeast strains (*cab1Δ*/*cab1*^N290I^ and *cab1Δ*/*cab1*^I291T^) lacking *CAB1* and expressing *cab1* alleles carrying pathological variants found in the human *PANK2* gene, have been shown to recapitulate all the major cellular phenotypes found in patient cells. These phenotypes include mitochondrial dysfunction, altered lipid metabolism, iron overload, and oxidative damage [24]. N290I and I291T substitutions correspond to missense mutations N500I and I501T in the *PANK2* gene found in some of the PKAN patients. 

The ease of handling of yeast and the amenability of this model organism to genetic manipulation makes it an ideal system to probe the role of human PanK enzymes in the regulation of cellular metabolism, in general, and in mitochondrial homeostasis, in particular. Herein, we provide evidence for the functional conservation of PanK cellular activity between humans and yeast using yeast as a model system. We show that expression of human PanKs leads to either complete (with PanK1 and PanK3) or partial (with PanK2 lacking a mitochondrial transit peptide) complementation of the growth and cellular defects caused by the G351S, N290I and I291T mutations in the *CAB1* gene. The functional complementation by human PanKs of yeast mitochondrial defects caused by altered Cab1 activity suggest that cytoplasmic expression of PanKs is sufficient for recovery of a normal mitochondrial function. The establishment of a yeast model of PKAN sets the stage for future investigations into the cellular, genomic, and metabolic complexity of the disease and could expedite the evaluation of new therapeutic strategies for the treatment of the disease in humans.

## 2. Results

### 2.1. Functional Analysis of Human PANK1, PANK2, and PANK3 in Yeast

Previous studies have shown that expression of a full-length human PanK2, with an intact mitochondrial targeting motif, does not complement the loss of the yeast *CAB1* gene [24]. 

**Figure 2:**
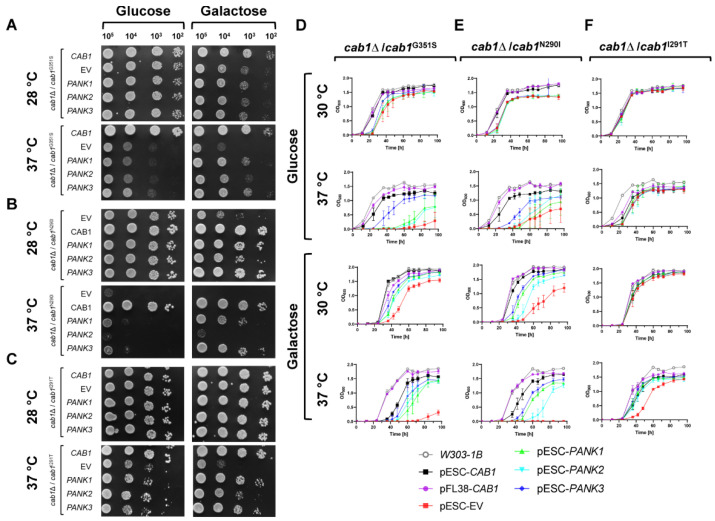
Complementation of the thermosensitive phenotype of *CAB1*-defective yeast strains by human PanKs. (**A**–**C**) Equal amounts of serial dilutions of cells (10^4^, 10^3^, 10^2^, 10^1^ cells/spot) from exponentially grown cultures of *cab1*Δ/*cab1^G351^*^S^ (**A**), *cab1*Δ/*cab1*^N290I^ (**B**), and *cab1*Δ/*cab1*^I291T^ (**C**) mutant strains carrying pFL38/*CAB1* and pESC-*URA3* vector containing the different h*PANK* gene were spotted onto SC medium supplemented with the indicated carbon sources and incubated at 28 °C and 37 °C. Yeast growth was monitored over time and images collected 2 or 3 days post-inoculation. (**D**–**F**) Growth rates of *cab1*Δ/*cab1*^G351S^ (**D**), *cab1*Δ/*cab1*^N290I^ (**E**), and *cab1*Δ/*cab1*^I291T^ (**F**) strains carrying the empty vector (EV), *CAB1* and human *PANK*s in liquid media on glucose or galactose at 30 °C vs. 37 °C (from top to bottom). Yeast cells were inoculated in minimal medium without uracil and tryptophan but containing either 2% glucose or 2% galactose at a cell density of 10^4^ cells/mL. Optical density measurements at 600 nm (OD_600_) were determined at the indicated time points. Data for liquid assays represent three biological replicates (*n* = 3) and the plotted graphs represent the average ± SEM.

To gain insights into the function of the active human *PANK*s, full length *PANK1* and *PANK3* as well as a truncated *PANK2*, lacking the mitochondrial targeting sequence (Pank2^mtmΔ^), were codon-optimized, using the yeast codon usage, and cloned into the pESC-*URA3* expression vector for expression as protein fusions with an N-terminal c-Myc tag. In these constructs, *PANK* expression is under the regulatory control of the *GAL1* inducible promoter, allowing transcription of the genes under galactose but not glucose conditions. The resulting plasmids were then introduced into yeast mutant strains lacking the chromosomal *CAB1* gene but expressing either *cab1*^G351S^ or the hypomorphic mutants *cab1*^N290I^, or *cab1*^I291T^. The ability of human PanKs to restore the cellular and metabolic defects of these mutants was examined (Figure 2).

Consistent with published data, both G351S and N290I substitutions caused a thermosensitive phenotype [24] and reduced growth on galactose both at 28 °C and 37 °C (Figure 2A,B,D,E). On the other hand, I291T showed a mild thermosensitive phenotype at 37 °C on both glucose [24] and galactose and this phenotype was further exacerbated in the presence of galactose at 37 °C (Figure 2C). In the *cab1*^G351S^ genetic background, all human isoforms rescued the growth phenotype of the mutant on galactose at 37 °C; however, the extent of the complementation varied between PanK isoforms with optimal complementation achieved with hPanK3. In the yeast strain harboring the *cab1*^N290I^ allele, which displays a more severe growth defect on galactose at 37 °C compared to the *cab1*^G351S^ mutant, expression of human PanK1 and PanK3 restored growth to levels similar to those seen in the *cab1*^G351S^ mutant background. However, expression of human PanK2^mtmΔ^ resulted in only partial complementation (Figure 2B). Finally, in the mutant strain carrying the *cab1*^I291T^ variant, which displays only a mild growth defect on galactose at 37 °C, expression of human PanKs restored growth to levels comparable to those of the wild-type strain (Figure 2C). Unlike the PanK2^mtmΔ^ isoform, expression of the full length human *PANK2* (harboring the mitochondrial transit peptide) in the hypomorphic mutants G351S and N290I failed to complementation their growth defect at 37 °C (data not shown).

**Figure 3:**
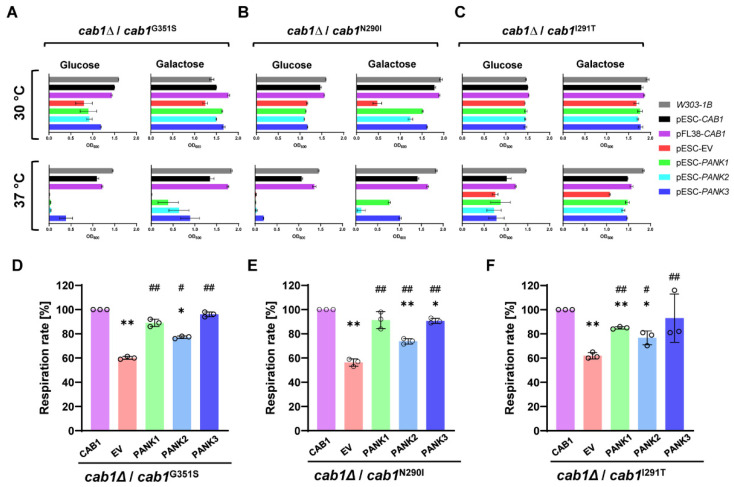
Cellular activity and respiration rate of mutant strains expressing human PANKs. (**A**–**C**). Representation of the mid-log phase growth of yeast strains cab1Δ/cab1^G351S^ (**A**), *cab1*Δ/*cab1*^N290I^ (**B**), and *cab1*Δ/*cab1*^I291T^ (**C**) carrying EV, *CAB1* and human *PANK*s from data shown in Figure 2. The bar graphs show the OD_600_ values of the strains expressing *PANK*s at the time point when the strains expressing the *CAB*1 wild-type allele were at the late log phase (36 h for glucose and 60 h for galactose conditions, respectively). (**D–F**) Oxygen consumption rate was measured in the same strains grown at 28 °C or 37 °C (especially for the strain expressing the *cab1*^I291T^ mutant allele) in SC medium without uracil and tryptophan and containing 2% galactose. OCR was normalized to the corresponding mutant strain transformed with the *CAB1* wild-type allele. OCR was conducted in triplicates (*n* = 3) and the plotted graphs represent the average ± SEM. *: *p* < 0.05 and **: *p* < 0.01 relative to mutant strain transformed with *CAB1* allele and #: *p* < 0.05 and ##: *p* < 0.01 relative to mutant strain transformed with the empty vector. Using ANOVA followed by Bonferroni’s post hoc test.

To further quantify the complementation level by human PanKs in these yeast mutants, the same assays were conducted in liquid media and cell growth was measured over a 96 h time period as shown in Figure 2D–F. Overall complementation efficiency by the human PanKs measured in liquid-based media was similar to that measured in solid growth media (Figure 3A–C). 

Quantitative analysis of the complementation efficiency of human PanKs was determined by comparing the growth rate of mutant cells harboring the human genes to that of cells carrying the empty vector or the wild-type *CAB1* gene under similar growth conditions as described in the Material and Methods. Expression of the human PanKs resulted in partial complementation of the growth defect of the *cab1*^G351S^ mutant at 37 °C on galactose, which was estimated to be ~22% (PanK1), ~36% (PanK2) and ~51% (PanK3) that of the complementation achieved with the wild-type *CAB1* (Figure 3A). In the yeast strain harboring the *cab1*^N290I^ allele, the estimates were ~47%, ~8%, and ~62 % for PanK1, PanK2, and PanK3, respectively, under the same conditions (Figure 3B). Although the *cab1*^I291T^ mutation has only a mild growth defect at 37 °C under galactose conditions, expression of human PanKs restored growth of the mutant to ~94% (PanK1), ~88% (PanK2), and ~94% (PanK3) that of the same strain carrying the wild-type *CAB1* gene (Figure 3C).

The ability of human *PANK*s to complement a null *cab1Δ* mutant was also determined by examining the loss of the *CAB1*-expressing plasmid in the yeast strain carrying a deletion of the chromosomal *CAB1* gene (data not shown). Although a few clones were detected, the rate of plasmid loss was found to be very low, suggesting lack of complementation of the null mutant. It is possible that the few clones detected were selected due to suppressor mutations. 

### 2.2. Complementation Analysis of Respiratory Activity Deficiency of cab1 Mutant Strains

Previous studies have demonstrated that strains expressing the hypomorphic alleles *cab1*^G351S^, *cab1*^N290I,^ and *cab1*^I291T^ exhibit mitochondrial dysfunction [24]. To assess whether *hPANK*s could rescue this phenotype, the oxygen consumption rate (OCR) of the mutants carrying an empty vector, yeast *CAB1* or the human *PANK*s was measured on galactose-based growth medium. As expected, optimal respiration rates were observed for strains carrying the wild-type *CAB1* allele whereas the mutants carrying the empty vector all showed reduced respiration rates (~60% that of the wild-type) (Figure 3D–F). In each of the three yeast backgrounds, expression of human *PANK1* and *PANK3* restored the respiration efficiency in the mutants to levels similar to those achieved with the wild-type allele (Figure 3D–F). Expression of the *PANK2^mtm^^Δ^* gene, however, resulted in partial restoration of the respiration rates with measured rates estimated at ~80% those of the mutants carrying the wild-type *CAB1* gene.

### 2.3. Human PanKs Complement Iron Susceptibility of Yeast cab1 Mutants

**Figure 4:**
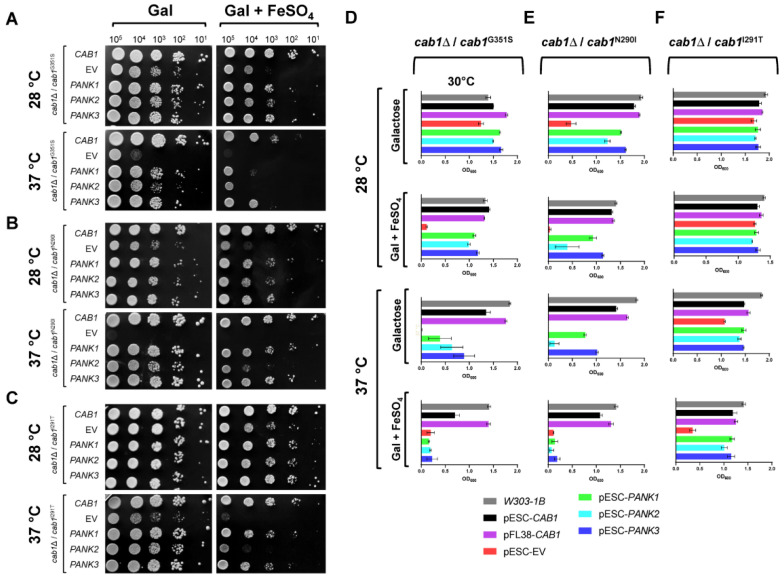
Human PANK1–3 complement iron sensitivity of yeast strains with defective PanK activity. (**A**–**C**) The iron susceptibility of cab1Δ/cab1^G351S^ (**A**), cab1Δ/cab1^N290I^ (**B**), and cab1Δ/cab1^I291T^ (**C**) yeast strains carrying an empty vector or either yeast CAB1 or human PANK genes was determined using plate-based assays. Cells were spotted at the indicated cell densities onto SC medium without uracil and tryptophan with 2% galactose in the absence or presence of 7 mM FeSO_4_. Cell growth was measured after 3 days of incubation at 28 °C and 37 °C. (**D**–**F**) Depit results of assays similar to those described in (**A**–**C**) but conducted in liquid culture with growth measured at 60 h post-inoculation. Liquid growth assays were conducted in triplicates (n = 3) and the plotted graphs represent the average ± SEM.

Iron dyshomeostasis is an important feature of PKAN disorder [11,18,19,24]. In yeast, iron accumulation results in increased susceptibility to FeSO_4_ [26]. Interestingly, the strain harboring the *cab1*^G351S^ or *cab1*^N290I^ mutant alleles showed greater sensitivity to this ferrous salt as well as iron accumulation [24]. To determine whether expression of hPanKs could also rescue the iron-sensitivity phenotype of the *cab1* mutants, growth assays were conducted in both solid and liquid galactose-based media lacking or supplemented with 7 mM FeSO_4_. As shown in Figure 4, the severe growth defect of all three yeast mutants (*cab1*^G351S^, *cab1*^N290I^, and *cab1*^I291T^) on FeSO_4_ was complemented by the expression of hPanKs under both permissive and restrictive temperatures. Expression of hPanK3 or hPanK1 resulted in complete rescue of the growth defect whereas that of h*PANK2*^mtmΔ^ resulted in partial complementation of this phenotype (Figure 4 and Figure S1).

### 2.4. Complementation of the Oxidative Damage of CAB1 Defective Yeast Mutants by Human PanKs

**Figure 5:**
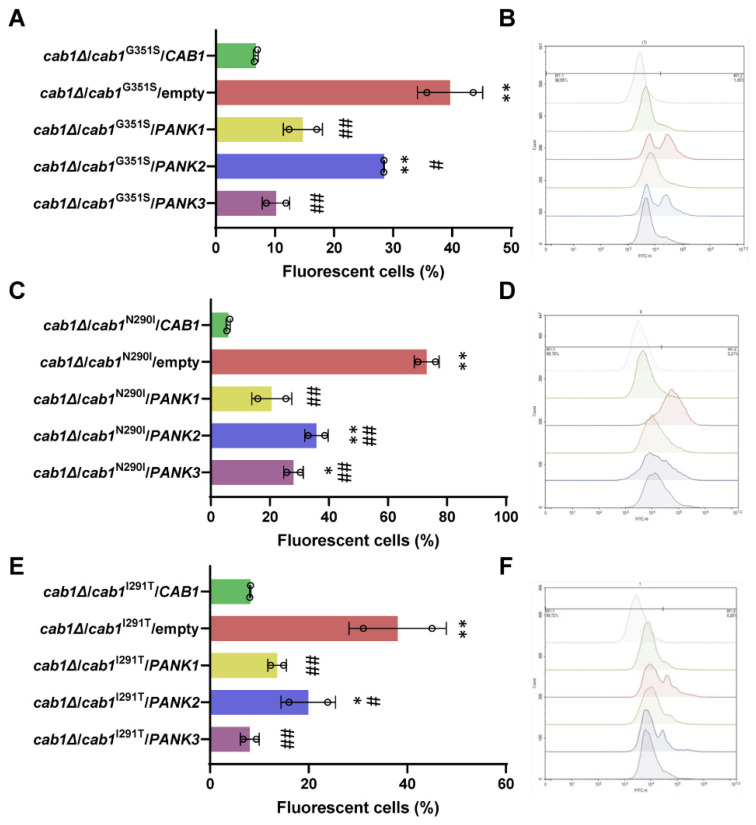
Analysis of reactive oxygen species (ROS) yeast PanK-deficient mutants expressing human PanK isoforms. ROS measurements were performed for *cab1*Δ/*cab1*^G351S^ (**A**), *cab1*Δ/*cab1*^N290I^ (**C**), and *cab1*Δ/*cab1*^I291T^ (**E**) yeast strains transformed with pFL38/*CAB1*, EV, and pESC-*URA3* vector containing different human PanK isoforms. ROS production was determined as the percentage of fluorescent cells using the DHR123 probe. Cytofluorometry analysis of the *cab1*Δ/*cab1*^G351S^ (**B**), *cab1*Δ/*cab1*^N290I^ (**D**), and *cab1*Δ/*cab1*^I291T^ (**F**) yeast strains transformed with either pFL38/*CAB1*, empty vector or pESC-*URA3* vector containing different human PanK isoforms following staining with DHR123 probe. Each curve represents the distribution of the measured events (counts) according to their fluorescence intensity expressed in a log unit. The grey spectrum represents background autofluorescence. The strains were grown in YP medium supplemented with 2% galactose at 28 °C. This assay was conducted in triplicate (*n* = 3) and the plotted graphs represent the average ± SEM. *: *p* < 0.05 and **: *p* < 0.01 relative to mutant strain transformed with *CAB1* allele and #: *p* < 0.05 and ##: *p* < 0.01 relative to mutant strain transformed with the empty vector.

It is well-described that iron accumulation is associated with an altered cellular oxidative state [27,28,29]. Accordingly, and as previously reported, the *cab1*^G351S^ and *cab1*^N290I,^ mutant strains exhibited a 3.5-fold increase in reactive oxygen species (ROS) compared to the parental strain. The mutants also showed an increase of malondialdehyde (MDA) content, suggesting lipid peroxidation and impairment of membrane function [24].Thus, ROS production was measured in all yeast mutant strains carrying human PanKs or the empty vector by cytofluorometric analyses using the fluorescent ROS indicator dihydrorhodamine 123 (DHR123) (Figure 5A–F). 

All yeast mutant strains carrying the empty vector showed high fluorescence signals, indicative of intense oxidative stress associated with the loss of PanK activity (Figure 5A–F). Conversely, cells expressing the wild type *CAB1* gene showed low levels of ROS indiating low oxidative stress levels. Interestingly, ROS content was significantly reduced by the expression of each of the hPanKs in all mutant genetic backgrounds (Figure 5A–F) with expression of human *PANK3* or *PANK1* in the mutants causing significantly greater reduction in oxidative stress compared to *PANK2^mtm^^Δ^*. 

### 2.5. Correlation between Complementation Ability and Human PanK Expression and Activity 

**Figure 6:**
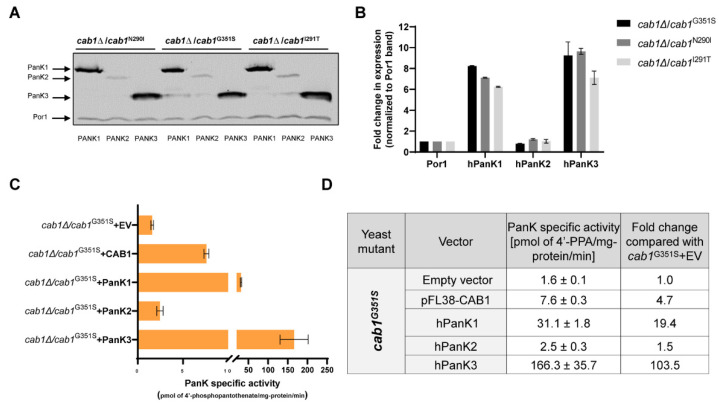
Expression levels and enzyme activity of hPanK1, hPanK2^mtmΔ^ and hPanK3 in yeast. (**A**) Western blot analysis using anti-Myc monoclonal antibody on extracts from the yeast mutant strains carrying human *PANK1*, *PANK2^mtm^^Δ^* and *PANK3*. (**B**) Quantification of the expression levels of human PanKs in yeast from (**A**) using Image Lab software. The signals were normalized to the control signal (α-Por1). Values are means ± standard deviation, *n* = 3. (**C**,**D**) Cellular pantothenate kinase was measured from fresh cell extracts obtained from *cab1*Δ/*cab1*^G351S^ yeast cells carrying either the empty vector, yeast *CAB1* or human *PANK* genes. Cells were grown at 30 °C in media containing 2% galactose. The activity assay measures the phosphorylation of D-[1-^14^C] pantothenate as described in Material and Methods.

To assess whether the degree by which human PanKs complement the growth defect, respiration rate and oxidative stress of yeast mutants altered in PanK activity could be linked to the level of expression of each enzyme, immunoblot analyses were conducted. Since human PanKs were expressed with a C-terminal Myc Tag, their expression in the *cab1*^G351S^, *cab1*^N290I,^ and *cab1*^I291T^ mutants following growth on galactose-based medium was monitored using an anti-Myc monoclonal antibody (Figure 6A,B). 

Whereas PanK1 and PanK3 proteins were strongly induced in these strains, the levels of PanK2^mtmΔ^ were very low in the three genetic backgrounds. As a control, the expression of the endogenous mitochondrial outer membrane protein porin (Por1) was also examined using antiPor1 monoclonal antibody and similar levels of this protein could be detected in all strains (Figure 6A,B). Consistent with these data, endogenous PanK activity in PanK-defective cells measured using D-[1-^14^C] pantothenic acid also showed correlation between levels of expression and enzyme activity. As expected, *cab1*^G351S^ cells harboring an empty vector exhibited low PanK-specific activity [5,25], whereas *cab1*^G351S^ cells expressing PanK1 or PanK3 showed comparatively higher PanK activity with PanK1 leading to 19.4-fold increase in the endogenous PanK activity of the mutant cells and PanK3 to ~104-fold increase in the endogenous PanK activity (Figure 6C,D). PanK2^mtmΔ^ expression in *cab1*^G351S^ cells resulted in only a modest (~1.5-fold) increase in the endogenous PanK activity. Together, these findings suggest that differences in expression levels and activity between human PanKs in yeast likely account for their ability to achieve complete or partial complementation of the cellular defects caused by the specific *CAB1* mutations.

## 3. Discussion

In this study, we provided the first evidence for the functional conservation of PanK function between humans and yeast. We found that growth and cellular and mitochondrial defects of yeast strains carrying *cab1*^G351S^, *cab1*^N290I^ and *cab1*^I291T^ mutated alleles of the pantothenate kinase gene *CAB1*, but not the null *cab1Δ* mutant, can be complemented by expression of active human PanK-encoding genes *PANK1*, *PANK2* and *PANK3*. In the case of human PanK2, initial attempts to express the full-length protein with its mitochondrial- targeting motif (MTM) failed to complement these defects, whereas a PanK2 isoform lacking this motif was functional in yeast although its expression could not reach levels sufficient to achieve full complementation. These studies are of particular importance as expression and regulation of *PANK2* are important factors in PKAN and the establishment of a reliable model to investigate the function of human PanKs would make it possible to express individual alleles found in patients and assess their cellular and metabolic impacts using yeast as a model system.

To date, the molecular mechanism underlying PKAN manifestation remains poorly understood. A simplistic model suggests that the loss of PanK2 activity leads to reduced levels of CoA, which in turn impacts a series of metabolic events that rely on this cofactor to achieve the correct cellular functions [30,31]. The validity of this model has, however, been challenged because CoA levels do not seem to be altered, if at all, in cells lacking PanK2 in vitro or mice [15,32,33]. Other molecular mechanisms have also been proposed including altered mitochondrial ACP, which controls the biosynthesis of fatty acids and protein lipoylation [15,33]. A better understanding of how loss of PanK2 causes PKAN may help in the development of optimal therapeutic strategies for this disease. Therefore, establishment model organisms, especially those that are amenable to high throughput genetic and cell biological analyses, could play a central role in advancing this knowledge. Mice, zebrafish (*Danio rerio*) and fruit flies (*Drosophila melanogaster*) have been used as models of PKAN and have helped answer important questions about the role of pantothenate phosphorylation in the onset of various physiological defects reminiscent of PKAN. Similarly, yeast, with its plethora of genetic and cell biological tools, represents an ideal system to probe the molecular and cellular mechanisms that lead to PKAN or PKAN-like phenotypes. In fact, the same yeast mutants used in this study have previously been shown to recapitulate several of the metabolic defects seen in PKAN [24].

Our data showed that PanK3 and PanK1 could be expressed at high levels in yeast and achieved the highest degree of complementation of all the phenotypes exhibited by the three yeast mutants defective in PanK activity, while a partial complementation was obtained by human PanK2 lacking a mitochondrial transit peptide. 

Immunoblot analyses also showed that the expression level of PanK2 is dramatically lower than that of PanK1 and PanK3, which may account for the differences in complementation of the cellular defects caused in yeast by different Cab1 mutations. It remains unclear why the expression of PanK2 is restricted by yeast cells. One possibility is that overexpression of PanK2 is detrimental to yeast cells through the modification of other unknown metabolites. This raises the possibility that this enzyme might play a role in other yet to be discovered metabolic pathways. This hypothesis could also explain the complex molecular and metabolic profile of PKAN. 

Unlike in *Drosophila*, where the localization of PanK to the mitochondria was crucial for optimal rescue of the PanK fbl mutant phenotype [22], the yeast Cab1 is a cytoplasmic enzyme [24,34] and optimal rescue is likely to be achieved with enzymes that are also expressed in the yeast cytoplasm. This could also explain the previously reported lack of complementation of yeast *cab1* null mutant and of hypomorphic mutants with the full-length PanK2, which carries the native mitochondrial targeting motif [24]. Research aimed to determine the localization of human PanKs in yeast and investigate the mechanism of complementation is warranted. Future molecular designs to express different forms of PanK2 as well as metabolomic analyses in yeast are warranted and may shed further light on the impact of PanK2 expression on yeast cellular metabolism.

Current therapeutic approaches for PKAN include activation of active cytosolic PanKs (PanK3 in particular) to compensate for the altered activity of the mutated hPanK2 [35], metabolite supplementation using intermediates in the CoA pathway such as 4’-phosphopantetheine [15] as well as the utilization of iron chelators such as deferiprone [36]. Moreover, extracellular CoA supplementation has been demonstrated to be an effective bypass treatment in cultured neuronal cells derived from PKAN patient fibroblasts [37]. Because none of these approaches has shown efficacy in PKAN patients, there is an urgent need to unravel the cellular and metabolic mechanism of the disease for the design of improved therapies. Yeast is thus an attractive model to study PKAN and may contribute to the discovery of an effective treatment for this disorder.

In summary, this study demonstrates complementation of pantothenate kinase deficiency in yeast by human PanK isoforms. These findings, which highlight the functional conservation in pantothenate phosphorylation across species, could set the stage for future biochemical and cell biological analyses in yeast to probe the effect of individual PanK2 mutations on cellular metabolism and mitochondrial activity. Strains carrying these mutations might serve as tools to evaluate new therapeutic strategies that reverse the phenotypes resulting from PanK loss of function. 

## 4. Materials and Methods

### 4.1. Yeast Strains, Media, and Plasmid Construction

Yeast strains, derived from W303-1B (Matα *ade2-1 leu2-3*, *112 ura3-1 trp1-1 his3-11*, *15 can1-100*), were grown on rich (YP) or minimal media (SC) with 2% carbon source as described before [24]. The human *PANK* genes, *PANK1* (Uniprot #Q8TE04), *PANK2* (Uniprot # Q9BZ23), and *PANK3* (Uniprot # Q9H999) were cloned into the pESC-*URA3* vector under the inducible *GAL1* promoter (GenScript Inc., Piscataway, NJ, United States), which results in expression of the proteins with an N-terminal Myc tag. The pESC-Empty vector (EV) was generated by releasing the *CAB1* insert by *Sal*I-*Hind*II digestion of pESC-*CAB1*, followed by filling-in of the overhang in the digested vector with T4-DNA polymerase and re-ligation of the vector. *CAB1* gene was expressed and evaluated with the pFL38*/CAB1* plasmid under the control of its native promoter and containing the *URA3* selectable marker gene [24]. All constructs, including the empty vector (EV) control, were transformed using the Li-Ac method [38] into the knockout strains harboring the defective alleles: *cab1*^G351S^ (*cab1Δ*/pFL39-*cab1*^G351S^), *cab1*^N290I^ (*cab1*Δ/pFL39-*cab1*^N290I^), or *cab1*^I291T^ (*cab1*Δ/pFL39-*cab1*^I291T^). Positive colonies were picked from SC-URA-TRP solid plates. 

### 4.2. Yeast Complementation and Iron Sensitivity Spotting Assays

Spot assay analysis was performed by spotting serial dilutions of the different strains on SC-U-W agar plates, supplemented with 2% galactose (inducing condition) or 2% glucose. When mentioned, the agar plates were supplemented with 7 mM FeSO_4_. Plates were incubated at 28 °C or 37 °C and captures were taken every 24 h. 

### 4.3. Oxygen Consumption Rate (OCR) and Reactive Oxygen Species (ROS) Measurements

The oxygen consumption rate (OCR) was measured at 30 °C in whole cells cultured for 18 h at 28 °C or for 16 h at 37 °C in SC-U-W medium supplemented with 2% galactose using a Clark-type oxygen electrode (Oxygraph System, Hansatech Instruments, King’s Lynn, UK) with 1 ml of air-saturated respiration buffer (0.1 M phthalate–KOH, pH 5.0) and 0.5% glucose. The reaction started by the addition of 20 mg of wet-weight cells as described [39]. Values for OCR were normalized to the dry weight of the cells.

ROS content was determined by flow cytometric analysis, using dihydrorhodamine 123 (DHR123; Sigma-Aldrich®, Darmstadt, Germany) that inside the cell can be oxidized by ROS (mainly H_2_O_2_), producing green, fluorescent R123 (excitation/emission spectra of 488/530 nm) detected by the fluorescence channel (FL-1) with a 530/30 nm band pass filter. For flow cytometry analysis, cells were pre-grown at 28 °C in SC liquid medium supplemented with 2% glucose and then inoculated in YP supplemented with 2% galactose. After 18 h of growth at 28 °C or 16 h at 37 °C, samples were loaded with 1.25 µg/mL of DHR123 for 2 h at 28 °C. At the end of the incubation time, cells were harvested (30 s at 14,000 rpm) and re-suspended in H_2_O. The fluorescence signal was quantified by a NovoCyte flow cytometer (NovoCyte^®^, ACEA Biosciences, Inc., San Diego, CA, USA). An unstained sample, without DHR123, was prepared as a negative control for each strain to set the threshold index, thus providing an auto-fluorescence background against which to measure positive fluorescence. For each sample, 10,000 cellular events were analyzed. Data achieved from flow cytometer were analyzed using NovoExpress software (NovoExpress^®^, ACEA Biosciences, Inc., San Diego, CA, USA). ROS generation was measured as the percentage of fluorescent cells (PFC) corresponding to cells that produced ROS-level increments of at least one log unit [40,41]. Values were normalized to the mutant strains harboring the *CAB1* wild-type allele.

### 4.4. Monitoring of hPanK Steady-State Expression in Yeast 

To determine the steady-state level of the hPanKs isoforms, all the mutant strains *Δcab1* expressing *PANK1*, *PANK2* or *PANK3* were pregrown in SC glucose 2% medium and then inoculated in SC medium supplemented with 2% galactose until 2–3 OD_600_ was reached at 28 °C or 37 °C depending on the mutated strain analyzed. Then, 10 OD_600_ cells for each strain were collected and total protein extraction was performed using the TCA method by chilling the cells in the presence of 20 mM NaOH, 0.5% β-mercaptoethanol, 650 µM PMSF and 25% TCA on ice. The protein extracts were then resuspended in 150 μl of Laemmli Sample Buffer at a pH of 6.8. An equal amount of protein suspension, corresponding to 1 OD_600_ of the original cell suspension was loaded on 12% SDS-PAGE and electroblotted on a nitrocellulose filter. The filter was incubated with mouse α-Myc (1:2000) and mouse α-Por1 (1:10,000) primary antibodies. Secondary antibody anti-mouse StarBright 700 (BioRad, Hercules, CA, USA, 1:10,000 dilution) was used to detect both primary antibodies. Fluorescent signals were revealed using Bio-Rad ChemiDoc Imagers and analyzed with Image Lab Software (Bio-Rad). The ratios between Myc and Por1 were calculated.

### 4.5. Liquid Yeast Growth Assays

For yeast growth assays, precultures of *S. cerevisiae* cells were grown overnight at 30 °C in SC-URA-TRP media (2% glucose). Cells were diluted to OD of 0.2 in the fresh media, grown 4 h to mid-log phase, and then harvested, washed, and adjusted in sterile water to OD_600nm_ = 1. Yeast cells were inoculated in polypropylene tubes at a final volume of 3.5 mL. The initial cell density was 10^4^ cells/mL in SC-URA-TRP media (2% glucose or galactose). A total of 6 mM FeSO_4_ was added to the media when needed. Cells were incubated at 30 °C and 37 °C and optical density (OD_600_) measurements were taken using a spectrophotometer. 

### 4.6. PanK Enzymatic Activity in Yeasts 

Pantothenate kinase activity assay was performed as previously described [1]. Briefly, the assay tracked the ATP-dependent conversion of D-[1-^14^C] pantothenate to phosphopantothenate and binding to the DEAE cellulose ion exchange filter. Cell-free extracts from yeast expressing human PanK proteins were obtained by homogenization using glass bead beating, followed by centrifugation at 1000 rpm for 5 min. The 40 μL enzyme reaction contained reaction buffer (100 mM Tris HCl, 2.5 mM MgCl_2_, 2.5 mM ATP, pH 7.4), D-[1-^14^C] pantothenate (2 nmol, 0.1 µCi), and 72 μg cell-free extracts. Protein content of the cell-free extracts was determined using the Bradford assay. The reaction was done at 30 °C for 10 min following the addition of 4 µL of 10% acetic acid to stop the reaction. The reaction mixture was spotted on a DE-81 filter (0.6mm in diameter) placed within a spin column with a 2 mL collection tube. Following 5 min incubation, the spotted filters were centrifuged for 20 s at 1000 rpm, washed twice with 1% acetic acid in ethanol, and collected for liquid scintillation spectrometry. 

## Data Availability

The data presented in this study are available in request from the corresponding author.

## Supplementary Materials

The following supporting information can be downloaded at: https://www.mdpi.com/article/10.3390/ijms24010435/s1.
