# Supplementary material for: Evidence for a Conserved Function of Eukaryotic Pantothenate Kinases in the Regulation of Mitochondrial Homeostasis and Oxidative Stress
Source: Int J Mol Sci. 2022 Dec 27;24(1):435. doi: 10.3390/ijms24010435 (PMC9820505; doi:10.3390/ijms24010435)
Supplement: Supplementary file 1 [file ijms-24-00435-s001.zip › ijms-2027084-supplementary.pdf]

Supplementary Materials

Evidence for a Conserved Function of Eukaryotic Pantothenate Kinases in the Regulation of Mitochondrial Homeostasis and Oxidative Stress

Camilla Ceccatelli Berti [1,†], Shalev Gihaz [2,†], Sonia Figuccia [1,2], Jae-Yeon Choi [2], Anasuya C. Pal [2], Paola Goffrini [1,*] and Choukri Ben Mamoun [2,*]

1 Department of Chemistry, Life Sciences and Environmental Sustainability, University of Parma, 43124 Parma, Italy
2 Department of Internal Medicine, Section of Infectious Diseases, Yale School of Medicine, New Haven, CT 06520, USA
* Correspondence: paola.goffrini@unipr.it (P.G.); choukri.benmamoun@yale.edu (C.B.M.); Tel.: +39-052-190-5107 (P.G.); +1-203-737-1972 (C.B.M.)
† These authors contributed equally to this work.

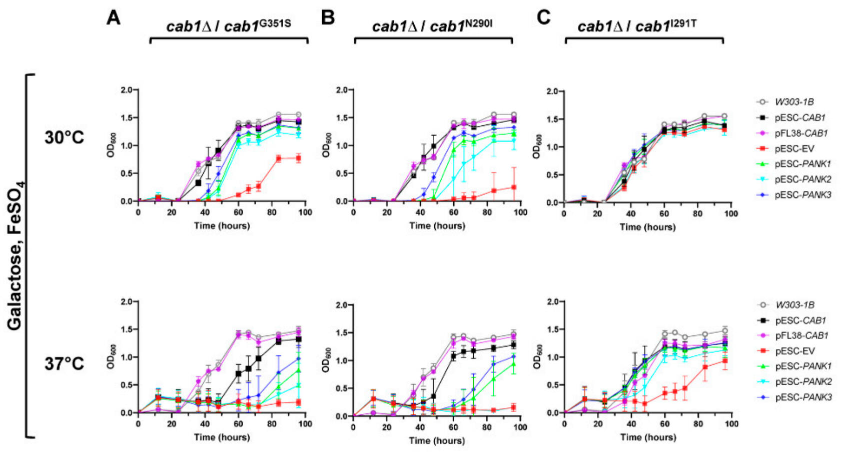

Figure S1. Iron sensitivity of PanK-deficient yeast strains expressing human PanKs. Growth curves of yeast strains *cab1*^{G351S}(A), *cab1*^{N290I}(B), and *cab1*^{I291T}(C) carrying an empty vector or expressing either the wild type yeast *CAB1* or human *PANK1*, *2*, or *3* were determined in liquid media containing 2% galactose at 30°C or 37°C (from top to bottom). Yeast cells were inoculated in tubes at a final volume of 3.5 mL at a density of 10^4cells/ml in SC-URA-TRP media (2% galactose) without or with 7 mM $FeSO_4$ and optical density (OD_{600}) was measured at the indicated time points. The assays were conducted in triplicates (n=3) and the plotted graphs represent the average ±SEM.
